# Collaborative Heterogeneous Mini-Robotic 3D Printer for Manufacturing Complex Food Structures with Multiple Inks and Curved Deposition Surfaces

**DOI:** 10.3390/mi16030264

**Published:** 2025-02-26

**Authors:** Karen Jazmin Mendoza-Bautista, Mariana S. Flores-Jimenez, Laisha Daniela Vázquez Tejeda Serrano, Grissel Trujillo de Santiago, Mario Moises Alvarez, Arturo Molina, Mariel Alfaro-Ponce, Isaac Chairez

**Affiliations:** 1 School of Engineering and Sciences, Tecnológico de Monterrey, Campus Guadalajara, Zapopan 45201, Mexico; karen.mendoza@tec.mx (K.J.M.-B.); mariana.s.flores@tec.mx (M.S.F.-J.); 2 School of Engineering and Sciences, Tecnológico de Monterrey, Campus Monterrey, Monterrey 64700, Mexico; a01423345@tec.mx (L.D.V.T.S.); grissel@tec.mx (G.T.d.S.); mario.alvarez@tec.mx (M.M.A.); 3 Forma Ciencia y Tecnologia, Forma Foods, Monterrey 64740, Mexico; 4 Instituto Tecnológico de Tláhuac III, Tecnológico Nacional de México, Tlahuac 13278, Mexico; amolina@tecnm.mx; 5 Institute of Advanced Materials for Sustainable Manufacturing, Tecnologico de Monterrey, Campus CDMX, Ciudad de México 14380, Mexico; mariel.alfaro@tec.mx; 6 Institute of Advanced Materials for Sustainable Manufacturing, Tecnologico de Monterrey, Campus Guadalajara, Zapopan 45201, Mexico

**Keywords:** additive manufacturing, food printing, curve-shaped printing, multi-ink food printer, collaborative robotics

## Abstract

The necessity of developing more realistic artificial food requires the aggregation of different biomaterials in an ordered and controlled manner. One of the most advanced methods for this is food printers reproducing additive manufacturing processes. This study presents a fully automatized 3D food printer leveraging collaborative Cartesian and multi-ink robotic systems to create complex food structures, with materials with different rheological settings using a screw conveyor configuration with controlled motion velocity. The developed food printer followed a formal mechatronic design strategy with fully functional instrumentation and automation systems. An adaptive controller was developed and implemented to regulate the coordinated operation of booth robotic devices, which are enforced by the G-code corresponding to the target food structure, leading to the necessary resolution. This device was tested with different commercial food inks to develop structures with complex shapes. The workability of the developed printer was confirmed by examining the food samples obtained using multiple materials, including creating different three-dimensional structures of a single complex food ink and creating simple structures made of different food inks with diverse structures that could yield a synthetic tissue that reproduces synthetic meat.

## 1. Introduction

The current conventional food chain is linked to environmental and human health damage [1], due to the large amount of resources consumed, greenhouse gas emissions, the high amount of food waste, the use of chemical fertilizers, genetic modification, and inefficient food storage [2,3].

The implementation of 3D printing technology in the food industry is gaining attention due to its advantages. For example, it can be used to design healthy and nutritional foods, introducing the required amounts of vitamins, proteins, fats, and fiber [4,5], which is currently of interest for the personalization of the diet in different social sectors, such as seniors, athletes, military, astronauts, or people with dysphagia [4,6], since food printing could also be used to improve the visual appeal of pure diets. In addition, it gives rise to the engineering of new materials, improving the typical food industrial process, achieving the world’s sustainable development goals, and reducing food waste and greenhouse gas emissions [1].

In this sense, 3D food printing can help in fulfilling the sustainable development goals (SDGs) established by the United Nations; specifically, it can help generate sufficient food for everyone at a low price, since it will significantly lower the cost of purchasing components and supplies [7]; it can enhance food nourishment and promote sustainable farming practices, since it can reduce waste in food manufacturing, enabling customization of food products, and enhancing nutritious value and food security [8]; it can also reduce the need for cooking and microwaving, thereby reducing the health risks related to exposing humans to toxic radio waves and carcinogens; and finally, it can promote the well-being of elderly people who are suffering from dysphagia, providing food that is easy to swallow, since different hydro-colloids can be easily added to improve aroma, taste, and texture [9,10].

Multi-material food printing technology is highly relevant in the meat market due to the need to mimic the complexity of animals in texture and components [11]. In this regard, multi-material printing can allow the manufacturing of multiple material samples with high quality, optimized texture, flavor, color, elasticity, and chewiness. However, it is commonly difficult to achieve this multi-material printing due to challenges such as aligning more than one type of food ink and creating a continuous seamless food filament with different materials [12]. Therefore, it is important to improve multi-material printing simultaneously, as proposed in the present work.

Despite the benefits of 3D food printing, it still poses key challenges, mostly regarding material printability, which limits mass production and the printing of complex shapes. When working with food inks, we deal with non-Newtonian fluids, mainly pseudoplastic and Bingham plastic behaviors [4,13]. Therefore, the printing system should be capable of extruding materials such as pastes, biopolymers, and hydrogels [14]. In [15], it was stated that a paste suitable for extrusion was in the Young’s modulus range from 10 to 141 kPa with a consistency index from 17 to 1500 Pa·s^n^. In [16], the authors simulated the flow of different gels based on pumpkin and potato, determining that an extrusion pressure below 200 kPa was sufficient to extrude the gels if the viscosity of 10^1^ to 10^3^ Pa·s was maintained at a shear rate of 10 s^−1^. To achieve an appropriate behavior and texture of rheology, it has been common practice to mix the main ingredient with additives such as starch, gelatin, xanthan gum, methylcellulose, and pectin [4,17]. However, this has been applied to just a few food inks such as chocolate, dough, rice flour, and vegetable pastes, while the extrusion of meat-based inks has not been explored mainly due to the absence of polysaccharides, which limits the setting mass and gelling capacity [5]. As has been noticed, extrusion strategies are the primary issue in novel 3D bioprinters. Three primary extrusion modalities have been explored to solve food printing hurdles: syringe-based extrusion by compressing the plunger, air-pressure extrusion, and screw-based conveyors [4,5,6]. The syringe-based method has been the most studied; however, it is more suitable for low-viscosity food like icings [4], while the screw conveyor has shown better performance for high-viscosity materials and is a continuous extrusion method [4,5].

Most of the available extrusion systems focus on using one material at a time and rely on adapting previous fused deposition modeling (FDM) 3D printers, as in [13], where they developed an interchangeable syringe–pump mechanism by transforming a conventional 3D FDM printer; the material was extruded-piston-driven through a 60 mL syringe. Another example is presented in [14], where they adapted an FDM printer with a real-time monitoring system using a camera, and a convolutional neural network was trained to select the correct parameters to obtain good resolution. Single extruder-free printers include the *Choc Creator V2.0 Plus (Choc Edge, London, UK)* with a 30 mL syringe [18], the *Model F5 commercial art pancake printer* that uses a delta configuration to deposit thin-layer foods, with a 1.2 L container [19], the *Pancakebot 2.0 (Pancake, New York, NY, USA)* that uses compressed air for extrusion [20], and the *Barilla* pasta printer (Barilla, Rome, Italy) [21]. Since maintaining material stability after deposition is also challenging, the authors of [22] proposed adding a blue laser (450 nm) to a Geetech i3 pro B 3D printer (HK Geetech Co, Honk Kong) to irradiate a starch suspension mixed with tartrazine. Other examples include two extruders that can print different materials. For instance, the *Procusini 3.0 Dual (Procusini, Frankfurt, Germany)* has a large print area and allows two materials to be loaded, up to 85 g, in closed syringes [23]. Another case is the *Discov3ry 2.0 extrusion system (Fabbaloo, Edmonton, Canada)*, a dual 60 mL syringe system that feeds the material into a spiral mixer connected to an Ultimaker 3D printer (Ultimaker, Utrecht, Países Bajos) [24]. In [5], a Prusa i3 3D printer (Prusa, Prague, Czech Republic) was adapted to hold two 20 mL syringes, allowing double extrusion. Finally, there are some examples of multi-material extrusion systems currently being explored, such as the *Fab@Home Model 3 (OpenSource, Cornell, NY, USA)* [25], which is an open-source multitool head printer, and the *F3D (Fusion3, New York, NY, USA)* [26], which presents a three-frame model mounted to deposit three paste-like materials and includes a heat source used to cook while the food is being printed.

Even though multi-material extrusion at the same time is of utmost necessity in food printing, most examples mentioned above only allow the deposition of one material at a time since the printhead should be rotated or moved in the Cartesian coordinates to change the material. In addition, current systems are designed for just one type of material, mostly chocolate or pastes; however, for printing more complex foods, for example, meat, a mixture of fats and cartilage is necessary; therefore, the extrusion system should allow the mixing of material with different rheological properties. Moreover, current examples require manual material refilling, implying stopping the printing and removing the cartridge, which limits mass production and makes it challenging to maintain a clean environment. Therefore, a more friendly refilling system is needed.

Most of the systems discussed are based on previous FDM 3D printers with all the usual mechanical characteristics. Thus, they are limited by printer restrictions, which have hindered the implementation of food printers with more degrees of freedom and the capability to print more complex constructs, such as curved or non-planar shapes. This research focuses on developing a 3D printer system with all the mentioned characteristics, including several extruders that can deposit viscous material simultaneously, allowing the possibility of constructing complex curved structures based on traditional computer-assisted design software.

The main contributions of this study can be summarized in the following list:

- A new 3D food printer based on the collaborative operation of a Cartesian and multi-joint manipulator, which can promote deposition in different spatial locations with diverse orientations of food inks.
- The proposed printer was fully designed, constructed, and automatized following a formal methodology that yields the possibility of extruding multiple materials simultaneously under controlled conditions.

This manuscript is organized as follows: Section 2 demonstrates the methodological strategy followed to develop the food printer with multiple extruders and a couple of robots. Section 3 presents the results corresponding to the food printer, detailing the mechanical, instrumentation, and automatization components of the proposed device. Section 4 presents a general discussion of the crucial achievements obtained in the current study. Section 5 closes the paper with some final conclusions and future work.

## 2. Design and Development of Collaborative Heterogeneous Mini-Robotic 3D Printer

This section presents the design and implementation strategy for the components needed to develop the additive manufacturing device. It details the mechanical, instrumentation, and automation elements. Moreover, the aspects of functionality and manufacturing validation are explained in detail.

### 2.1. Design Requirements for the Food Printer

The conditions needed to develop artificial food products imply that multiple materials can be extruded simultaneously, with the possibility of depositing such materials on curved surfaces.

The following operational requirements are proposed to satisfy the crucial aspects of developing the proposed printer.

- Hyper-redundant configuration for the robotized section of the 3D printer to deposit extruded materials on curved surfaces.
- Multiple operative channels to extrude several materials simultaneously with an independently controlled flow.
- Regulated motion for all the actuators in the hyper-redundant configuration commanded by traditional G-code instruction set.
- Innocuous materials for developing the extruder containers.
- Space resolution of 0.002 m in the x-y plane and 0.001 m in the z plane.
- Motion velocity of the extrusion system of 0.2 m/s in all three motion axes.
- Material extrusion flow is regulated according to the rheological conditions of food inks.
- Minimum volume in the extruder device.
- Graphic user interface.

Table 1 summarizes the key details of the technical aspects considered in the food printer presented as requirements for the proposed device.

### 2.2. Mechanical Design of the Food Printer

**Figure 1:**
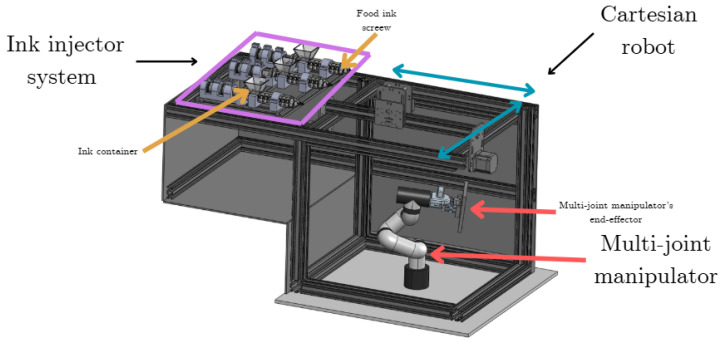
General mechanical configuration of the food printer instrumenting both robotic systems, including the Cartesian and the manipulator devices and the set of injector–extruder systems based on screw conveyor configuration.

Figure 1 depicts the printer’s 3D mechanical configuration, considering the general distribution of the Cartesian robotic device and the multi-joint robotic manipulator in the right section placed the first at the top and the second at the bottom of the printing chamber, configuring a collaborative robotic configuration.

On the left-hand side of the same figure, the set of three extruders is depicted, providing the option to extrude multiple food inks. Each extruder is formed with a container for the selected ink, an actuated screw system that promotes the extrusion of the selected material, and a DC motor that mobilizes the screw section with constant angular velocity.

The two collaborative robotic systems give the extruded material’s obtained shape a degree of freedom and smoothness that overcomes the possible forms obtained with standard food printer configurations. Based on the combined eight-degree-regulated freedom, it can drive a higher precision and reliability printhead while producing more complex extruded shapes.

The collaborative robotic configuration enforces the two proposed mobile frames: one moving along the x-y plane at the top of the proposed platform and the second placed at the end effector of the robotic manipulator.

The printer has two robotic systems generating a total of eight degrees of freedom, allowing it to drive a printhead with greater precision and reliability while producing more complex extruded shapes. The robotic configuration has a Cartesian system with 2-degrees-of-freedom: one that moves along the x-y plane at the top of the platform, where the end effector is the extruder of the bio-inks. The second system is a 6-degree-of-freedom robotic arm whose end effector contains the base where the bio-ink is printed.

Cartesian spatial motion is achieved by moving two stepper motors that form the Cartesian robotic section, including an X-axis guide rail and two parallelized Y-axis guide rails. This system is controlled by an OpenBuilds Inc. board, whose movements are based on the G-code. The X and Y axes are 400 mm, with a resolution of 0.5 mm in each of the axes. The robotic arm simultaneously moves its six joints, giving both the necessary height and the reach areas in the XY space that allow the printing of complex structures. This robotic device is a MyCobot M5 280 (Elephant Robotics Inc., Shenzhen, China). The joints 1–5 of the robotic arm can rotate 165°, and its sixth joint has a rotation angle of 165°. It generates a maximum working height of 400 mm, a working radius of 280 mm, and a motion repeatability of ±0.5 mm.

The developed configuration of two simultaneous devices leads to a maximum forming size of the suggested multichannel 3D bioprinter of 600 mm × 300 mm × 200 mm, and a maximum rotation with respect to the non-inertial frame of the end effector in the robotic manipulator of ±85°, ±5° and ±85° according to the standard sequence of axes (XYZ).

The spatial resolution is 0.5 mm × 0.5 mm × 0.1 mm in the spatial coordinates, with a repeatability of ±0.1 mm in the x plane and ±0.05 mm in the Z axis. The maximum Cartesian motion velocity was 10 mm/s for the X-Y plane. Complementarily, the robotic manipulator allows its end effector to move with a maximum displacement velocity of 0.1 mm/s × 0.1 mm/s × 0.1 mm/s in its corresponding x-y-z plane and 0.1 rad/s × 0.1 rad/s × 0.1 rad/s measured from the non-inertial frame at the end effector.

The dual Cartesian–manipulator configuration allows a precise layer-by-layer deposition of food ink. Given that the z axis has a vertical travel of 0.1 m, a precision of ±0.1 mm, and a repeatability of ±0.05 mm, the z layer of the deposited material can be accurately controlled.

A set of three injectors were included to mobilize the selected food inks from an individual reservoir to the extruder placed at the end effector of the Cartesian robot through flexible polymeric pipes. Each injector consists of an ink container, an internal screw, and a motor that provides the motion energy.

The container and screw sections were designed using computer-assisted design software (SolidWorks^*TM*^, 2023, Dassault Systemes SolidWorks Corporation, Paris, France) and developed using additive manufacturing strategies. A masked stereolithography additive manufacturing device was used to produce both pieces, the container and the screw. A FormLabs form 4 printer (FormLabs, Somerville, MA, USA) was used to manufacture these sections. The process conditions were vertical alignment, 0.050 mm layer thickness, and complete base support (0.55 mm contact size). The manufacturing material was Clear Resin V5 (FormLabs, Somerville, MA, USA) from the same company, which has the following main characteristics: ultimate tensile strength: 60 MPa; tensile modulus: 2750 MPa; elongation at break 8%; flexural strength: 105 MPa; flexural modulus: 2700 MPa.

The extruder section met the configuration in the studies presented in [27,28].

**Figure 2:**
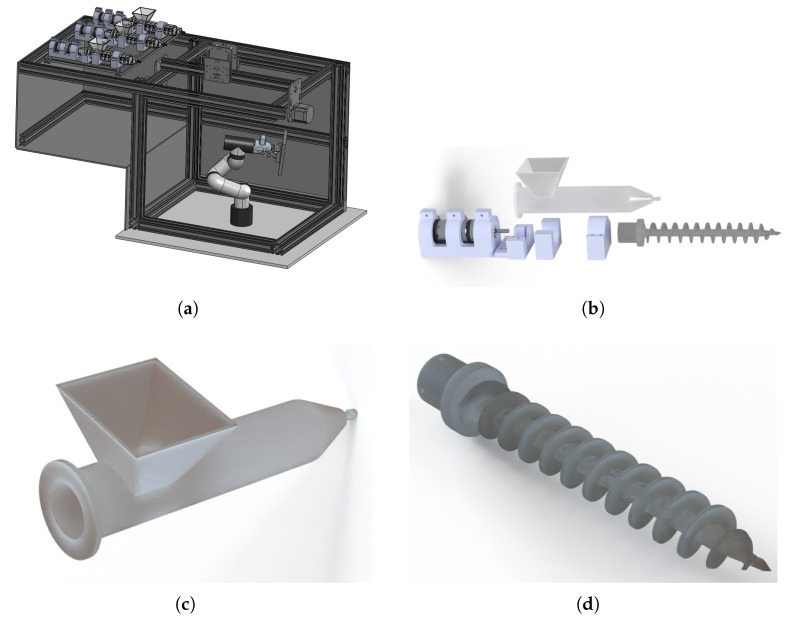
Composition of figures that detail the mechanical sections of the food printer. (**a**) Detailed view of the coordinated robotic configuration based on Cartesian–manipulator devices. (**b**) Exploded view of injector configuration. (**c**) Detailed view of food ink container in the injector device. (**d**) Detailed view of food ink screw in the injector device.

All mentioned components were supported on an aluminum-based preformed rail structure, configured to support mechanical sections of robotic systems, extruders, injectors, and electrical instrumentation elements (Figure 2).

### 2.3. Instrumentation of the Food Printer

The proposed food printer consists of three crucial stages that are electrically and electronically instrumented: the Cartesian device, the robotic manipulator, and the set of injectors.

The Cartesian device is mobilized using stepper motors (NEMA-34) connected to the section that transports extruders. Motion is transmitted through belts and pulleys in both directions, the x and y axes. A Black Box-X32 Driver operates the electrical signals that adjust the motors’ number of steps and motion direction. This device can handle three drivers that handle stepper motions in the x-y axes for Cartesian. In addition, the limit switches to preventing movement beyond admissible boundaries are also attached to this device. The robotic manipulator was controlled and energetically supplied with the proper systems of Elephant Robotics Inc., Shenzhen, China.

The screws inside injectors are attached to direct current (DC) motors (ServoCity planetary gear motor SKU638310, 20 RPM, 12 volts) that are regulated using a pulse-width-modulated (PWM) strategy with an operating frequency of 10 KHz and a 10-bit resolution. A set of DC-AC power BTS7960 (43 Amp max at 12 volts nominal) converters driven by a microcontroller modify the active pulse width based on the relative ratio between food inks that conform to the expected final product.

**Figure 3:**
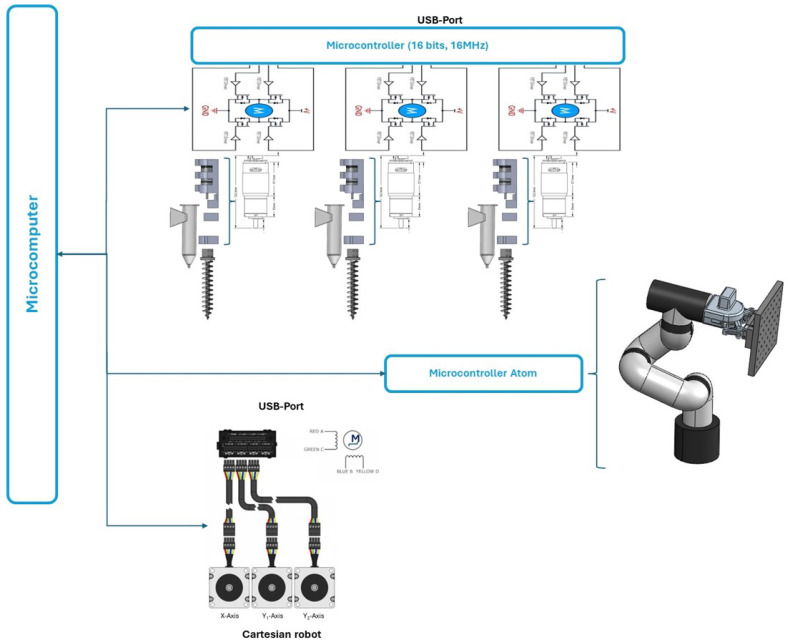
Full set of electrical instrumentation configurations used for regulating the printer activity.

Figure 3 demonstrates the general configuration of the electrical instrumentation used in the proposed food printer considering the three sections described above.

### 2.4. Automation Strategy for Controlling the Food Printer

**Figure 4:**
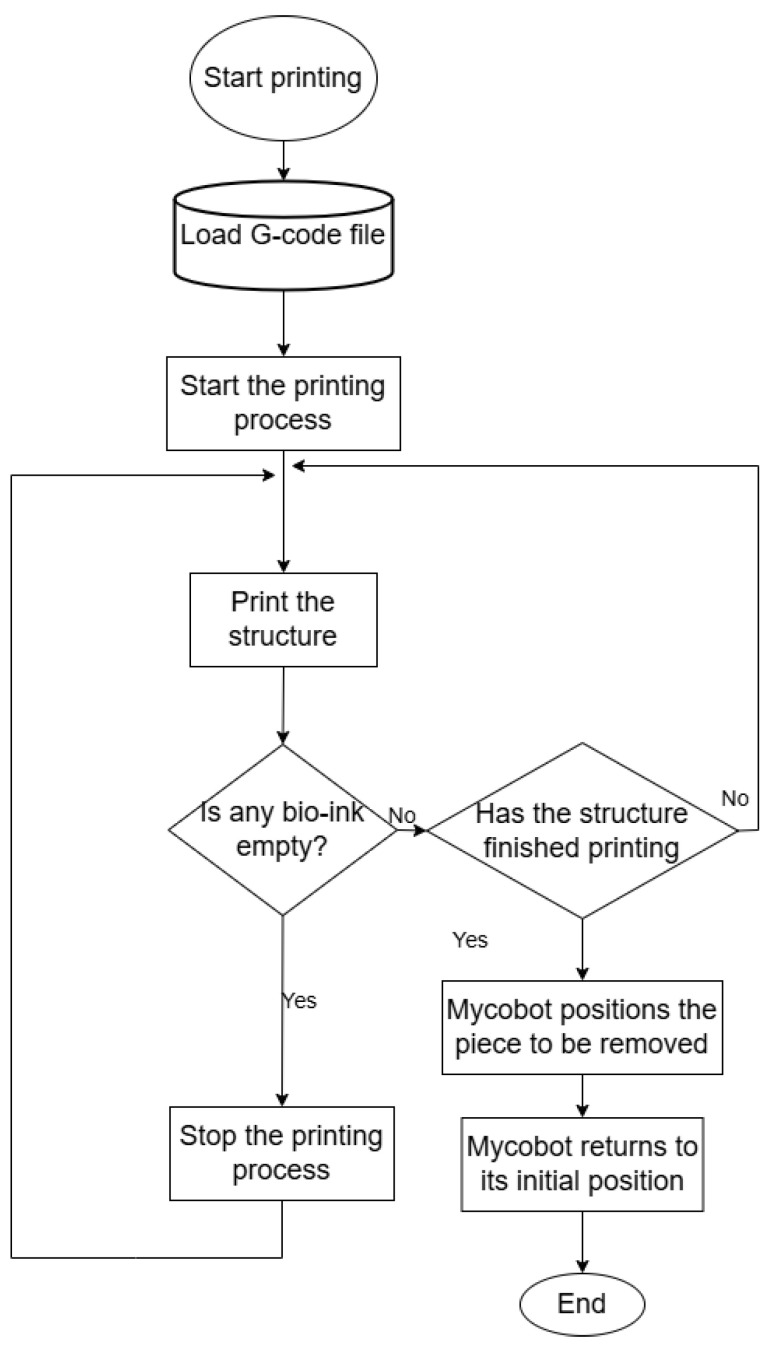
Operation diagram of the printer control flow considering the proposed three instrumented sections.

The control system of the multichannel 3D printing system consists of three subsystems: programmable multi-axis controller (PMAC) motion control, and electrical extrusion control. As a result, in addition to the control system’s hardware design, a host computer program must be built to operate the subsystems mentioned above effectively and precisely, beginning with a source stl (transferred to a G-code) file corresponding to the desired design. Therefore, a block diagram was developed to visualize the printer control flow (Figure 4).

Currently, the program design must be separated into the following three stages: The first stage is the creation of host computer software based on a fully operational minicomputer (Intel 12th Alder Lake N95, 3.4 GHz processor, GPU UHD graphics processor, 16 GB DDR4, 512 GB SSD, Intel, Santa Clara, CA, USA) to ensure that the input information and parameters of the man–machine interface may be accessed at any time.

The second stage would ensure that the communication channel between the computer mentioned above and the three low-level operative sections can be opened or closed at any time: a 16-bit microcontroller that regulates extruder operation using a PWM regulation strategy, the internal MyCobot computer, Elephant Robotics Inc., Shenzhen, China. that regulates the manipulator configuration, and the drive box that regulates Cartesian operation.

The third stage would allow for the interpretation and transmission of the resulting G-code to the manipulator and Cartesian devices, OpenBuilds, Phoenix, AZ, USA. The same user interface may be used to view the current configuration state of each robotic component and the motion data sent to the PMAC. Thus, the generated program and control operation technique are combined to create a fully integrated multichannel 3D printing system.

The automation strategy proposed in three stages requires additional motion coordination between the end effectors of both robotic systems: Cartesian and manipulator. Cartesian motion depends on the belt-pulley transmission system as a function of G-code. Hence, the manipulator provides the z coordinate and orientation. Hence, the coordinate dynamics of the Cartesian and manipulator robots induce a control problem that implies the design of a nonlinear state feedback controller that stabilizes the tracking error for all joints that correspond to perform the motion between both actuators with a predefined distance and orientation of the manipulator’s end effector.

For this study, we introduce the position of the selected end effector in the Cartesian device represented by the vector ${\vec{r}}_{C}\in R^{3}$, ${\vec{r}}_{C}={\left[x_{C},y_{C},z_{C}\right]}^{⊤}$, and the manipulator’s end effector position represented by the vector ${\vec{r}}_{M}\in R^{3}$ with coordinates corresponding to ${\vec{r}}_{M}={\left[x_{M},y_{M},z_{M}\right]}^{⊤}$. This selection of coordinates is fitted to the composite configuration of the proposed printer.

The evolution of $q_{a}$ satisfies the following differential equations [29,30] given the application of the Euler–Lagrange theoretical results:

(1) $$\begin{array}{c} \frac{d}{dt}q_{a}=q_{b}\\ \frac{d}{dt}q_{b}=f_{q}\left(q_{a},q_{b}\right)+g_{q}\left(q_{a}\right)u_{q}+\xi_{q}\left(q_{a},q_{b},t\right) \end{array}$$

The nonlinear function $f_{q}:R^{6}\times R^{6}\to R^{6}$ represents the internal dynamics of the manipulator systems including Cartesian and robotic arm elements. The input-associated function $g_{q}:R^{6}\to R^{6\times 6}$ characterizes the effect of motor torques on robot dynamics, either by using the screw motion transfer system or the direct application on each joint.

The function $f_{q}$ is locally Lipschitz concerning the corresponding arguments, given that we need the model used for control design to represent the actual robot dynamics. The matrix function $g_{q}$ is continuous, bounded, and invertible. The initial conditions for $q_{a}$, are $q_{a,0}$ and $q_{b,0}$, respectively. For all valid initial conditions and all essentially bounded controls $u_{q}\in U_{q}\subset R^{6}$ (representing the torques exerted by all actuators), there is a unique coordinated system solution (1) denoted by *q* for the corresponding dynamics of the manipulator.

The term $\xi_{q}:R^{6}\times R^{6}\times R^{+}\to R^{6}$ defines the effect of the admissible modeling uncertainties and external perturbations that modify the dynamics of the selected robotic section, the manipulator or the Cartesian component.

Consider the relative angles of the end effector $\vec{\theta }$ for the manipulator concerning the inertial framework given by a three-dimensional vector $\vec{\theta }={\left[\theta_{x},\theta_{y},\theta_{z}\right]}^{⊤}$.

Given that the system presented in (1) can be represented for control design purposes,

(2) $$\frac{d}{dt}x=f\left(x,u,\xi \right)$$

Here, $x=\left[q_{a}^{⊤},q_{b}^{⊤}\right]$ ($x\in X=X_{q,a}⊕X_{q,b}$), $u=\left[u_{q}^{⊤}\right]$ ($u\in U=U_{q}$), $\xi =\left[\xi_{q}^{⊤}\right]$ξ∈Ξ and the function *f* are formed with the appropriate functions on the right-hand side of (1). The initial conditions of (2) are $x_{0}\in X$.

According to the results presented in [31], the dynamic system presented in (1) is well posed considering that (a) f(x,u,0)∈TX where TX the tangential space of *X* for all x∈X, (b) f(·,u,0) is continuous for each u∈U, (c) f(x,·,0) is continuous for each x∈X, (d) for each f(·,·,ξ) is continuous for each ξ∈Ξ, and (e) for each u∈U and $x_{0}\in X$, there are δ>0 and locally integrable functions $L,L_{0}:R^{+}\to R^{+}$ such that

(3) $$\begin{array}{c} {∥f\left(x_{a},u,\xi \right)-f\left(x_{b},u,\xi \right)∥}_{X}\leq L\left(t\right){∥x_{a}-x_{b}∥}_{X}\\ {∥f(x_{0},u,\xi )∥}_{X}\leq L_{0} \end{array}$$

Supposing that system (1) is well posed, then its bounded maximal solutions are global, which satisfies the boundedness-implies-continuation property (BIC). Based on the well-defined system (1), the tracking control problem can now be formulated [32], which implies that we can ensure the tracking of the required motion for the printing procedure.

Problem definition on the coordinated motion of robotic components. Define the variable $\Delta_{r}\in R^{3}$ that characterizes the relative distance between the end effectors of both robotic devices, that is $\Delta_{r}={\vec{r}}_{C}-{\vec{r}}_{M}$. Also, consider that the variable $\Delta_{\theta }={\vec{\theta }}^{*}-\vec{\theta }$ defines the relative difference between a vector of reference angles ${\vec{\theta }}^{*}\in R^{3}$ and the ones measured for the manipulator. The problem considered in this study is to design the control action for the manipulator section $u_{q}\in R^{6}$ of the composite robot such that $\Delta_{r}$ and $\Delta_{\theta }$ satisfy

(4) $$\begin{array}{c} ∥\Delta_{r}∥\leq \beta_{M,r}+D\;∥\Delta_{\theta }∥\leq \beta_{M,\theta } \end{array}$$

Here, $D\in R^{+}$ is the desired distance that must be kept between both end effectors. Inequalities in (4) should be satisfied $\forall t\geq T_{M}$, where $T_{M}>0$ is the convergence time for manipulator operation. The control design problem should consider the coordinate restrictions for both robots in the composite structure.

Taking into account the problem for $\Delta_{r}$, $\Delta_{\theta }$, observe that the manipulator’s relative position is specific to the Cartesian device’s end effector. In the three-dimensional space $\Omega_{M}$, an acceptable area is defined by location ${\vec{r}}_{C}$ and admissible distance *D*. The unique configuration of the manipulator at a given time *t*, ${\vec{r}}_{M}^{*}\left(t\right)$, is determined once the reference angles $\theta_{r}^{*}$ are given.

Following the provision of the time-varying vector ${\vec{r}}_{M}^{*}\left(t\right)$, the corresponding time-varying vector for the articulations in the manipulator described by $q_{a}^{*}\in R^{6}$ is defined utilizing the inverse kinematics of the chosen manipulator. Based on this vector, the time-varying vector $q_{b}^{*}\in R^{6}$ may be found by direct differentiation or employing robust-exact differentiators. Assume that $q_{b}^{*}$ has a known derivative (that is, $d_{q}^{*}$) or can be computed online using any of the methods described above. It should be noted that the admissible subspace is taken into account while defining acceptable joint configurations for the manipulator. Using the newly introduced reference trajectories $q_{a}^{*}$ and $q_{b}^{*}$, the manipulator’s tracking error is developed. The dynamics of the tracking errors may be found by taking into account the following definitions for the tracking errors in the joint space: $\Delta_{q,a}=q_{a}^{*}-q_{a}$ and $\Delta_{q,b}=q_{b}^{*}-q_{b}$.

(5) $$\begin{array}{c} \frac{d}{dt}\Delta_{q,a}=\Delta_{q,b}\\ \frac{d}{dt}\Delta_{q,b}=d_{q}^{*}-f_{q}\left(q_{a},q_{b}\right)-g_{q}\left(q_{a}\right)u_{q}-\xi_{q}\left(q_{a},q_{b},t\right) \end{array}$$

When implementing the tracking trajectory controller, it is always important to observe the motion restrictions of each joint; the related constraints for the tracking errors must be evaluated and considered in the control design a priori. It is feasible to estimate the valid areas for the tracking errors given the motion limitations placed on each of the manipulator’s joints. The following disparities can be used to describe these areas [33,34].

(6) $$\begin{array}{c} \Delta_{q,a,i}^{-}<\Delta_{q,a,i}<\Delta_{q,a,i}^{+}\\ \Delta_{q,b,i}^{-}<\Delta_{q,b,i}<\Delta_{q,b,i}^{+} \end{array}$$

Here, $\Delta_{q,a,i}\in R$ is the ith component of $\Delta_{q,a}$ and $\Delta_{q,b,i}\in R$ is the ith component of $\Delta_{q,b}$. Also, the following variables are proposed $\Delta_{q,a,i}^{-}=q_{a,i}^{-}-q_{a,i}^{+}$ and $\Delta_{q,a,i}^{+}=q_{a,i}^{+}-q_{a,i}^{-}$ for the position variable. Likewise, $\Delta_{q,b,i}^{-}=q_{b,i}^{-}-q_{b,i}^{+}$ and $\Delta_{q,b,i}^{+}=q_{b,i}^{+}-q_{b,i}^{-}$ are considered for the derivative of the tracking error variables.

For the purposes of this study, the following relations are taken into account $\Delta_{q,a,i}^{-}=-\Delta_{q,a,i}^{+}$ and $\Delta_{q,b,i}^{-}=-\Delta_{q,b,i}^{+}$. This condition is not complex to fulfill given that the reference position of all articulations in the robotic arm can be freely adjusted.

Notice that the convergence of the tracking error (TE) combined with the fulfillment of conditions given in (6) imply that the states of the robotic manipulation must be restricted by the estimated upper and lower bounds, respectively. A constrained adaptive controller based on the Barrier Lyapunov function technique is proposed to avoid motion constraint transgression and solve trajectory tracking between the manipulator and the set of reference trajectories. Following the ideas related to Barrier control formulation, the controller $u_{q}$ is proposed to satisfy the following structure [35,36]:

(7) $$\begin{array}{c} u_{q}=g_{q}^{-1}\left(q_{a}\right)\left(d_{q}^{*}-K_{p}\Delta_{q,a}-K_{d}\Delta_{q,b}-f_{q}\left(q_{b},q_{a}\right)-K\Delta_{q}\right) \end{array}$$

where *K* is the state-dependent gain used to include the effects of state restrictions proposed as constraints for the angular displacements in all joints of the robotic manipulator.

The matrices $K_{p}$ and $K_{d}$ are selected in such a way that the following matrix is Hurwitz:

(8) $$A=\left[\begin{array}{cc} 0_{6} & I_{6}\\ K_{p} & K_{d} \end{array}\right]$$

The dynamics of state-dependent gain *K* can be found as a solution to the following differential equation:

(9) $$\begin{array}{c} 2\gamma \frac{d}{dt}K+\left(\frac{d}{dt}\gamma +\alpha \right)\tilde{K}+2P\Delta \Delta^{⊤}=0. \end{array}$$

Here, $\tilde{K}=K+K_{*},$ with $K_{*}$ is selected such that $A_{K}=A-K_{*}$ is a Hurwitz matrix, the state-dependent function γ must be positive for all the states and must take into account the effect of state constraints, and α is a positive scalar. The dynamic evolution of γ satisfies

(10) $$\gamma =\sum_{i=1}^{6}{\left(\frac{{\left[\Delta_{q,a,i}^{+}-\Delta_{q,a,i}-\varepsilon \right]}_{+}}{\Delta_{q,a,i}^{+}}\right)}^{2}\times {\left(\frac{{\left[\Delta_{q,a,i}-\Delta_{q,a,i}^{-}+\varepsilon \right]}_{+}}{-\Delta_{q,a,i}^{-}}\right)}^{2}.$$

Here, ε>0. The cutting function ${\left[\alpha \right]}_{+}$ meets the following definition:

(11) $${\left[\alpha \right]}_{+}=\left\{\begin{array}{cc} \alpha , & \alpha \geq 0,\\ 0, & \alpha <0. \end{array}\right.$$

In the dynamic form of the gain given in (9), the matrix *P* is a solution of the following matrix inequality [37]:

(12) $$\begin{array}{c} Ric_{A}\left(P\right)=PA_{K}+A_{K}^{⊤}P+PRP+Q<0,\\ A_{K}=A+K_{*},\\ R=L^{-1}\Lambda_{1}^{-1}{\left(L^{-1}\right)}^{⊤}+\Lambda_{2}^{-1},\;Q=>0 \end{array}$$

where *L* is the positive definite matrix of inductances, $\Lambda_{1}=\Lambda_{1}^{⊤}>0$, $\Lambda_{2}=\Lambda_{2}^{⊤}>0$.

The control action used in this study has been considered in [38,39]. The formal convergence proof of the mentioned controller can be consulted in detail within the references mentioned above. Notice that this control action is applied continuously, considering that the distance and relative orientation between both end effectors must be satisfied to deploy the food ink from the extruder to the collecting surface, which has a curved shape. The implementation of this controller also enforces the effective interaction between both robotic devices, which provides a novel operative condition for the regular operation of food printers.

### 2.5. Instrument Validation

A set of printer evaluations were processed. This study used several bio-inks based on a mixture of vegetable-based food ink composed of some other materials, a regular condition in developing synthetic meat. Concerning ink characterization, we completed the rheological characterization of inks based on synthetic meat. This study presents the results corresponding to the characterization of the selected ink that we have defined as suitable to continue the proposed experiments and its comparison with a reference food ink based on 20% pea protein. The characterization included the kinematic and dynamic viscosity, shear stress, and complex modules. A rheometer (MCR520, Anton Paar, Austria) with parallel plate geometry PP25 (25 mm diameter) and a 1 mm gap was considered. During the extrusion procedure, shear-thinning properties were analyzed, and the Ostwald–de Wale power law model $\left(\eta =K_{f}\cdot \gamma_{f}^{\left(n-1\right)}\right)$ was considered to describe the correlation between the apparent viscosity of the food ink and the shear rate [40]. At the same time, η, $\gamma_{f}$, $K_{f}$ and *n* are the apparent viscosity (η), shear rate ($\gamma_{f}$), consistency coefficient ($K_{f}$), and power law index (*n*). Recovery properties were assessed using the thixotropy experiment, which used three different shear rates (1 s^−1^, 10 s^−1^, and 100 s^−1^) to alter viscosity. The high-frequency sweep from 1 to 100 rad/s within the linear viscoelastic range (LVR) of $1\times 10^{-3}$ was used in the dynamic rheology test to analyze the connection between the angular frequency (ω) and the complex modulus (*G*).

The suggested 3D food printer and Repetier-Host V3.2.1 software were used for printing. Briefly, the formulations were deposited in injectors and coupled to a printhead containing a 22G nozzle [41]. A G-code for 1×1 cm perimeter squares was written manually. The squares were extruded to a flow rate of 20 µL min^−1^ and three different velocities (40, 60, 80 mm min^−1^). The G-codes for the 3D-printed structures were obtained from Gleadall [42], and the dimensions were custom-shaped using PrusaSlicer 2.5.2 software.

In addition, three food inks provided by Forma-Food company, Monterrey, MExico were used to validate each injector–extruder system on the printer: a vegetable-based artificial meat, synthetic vegetable fat, and connective-like tissue that aggregates the other two. The composition of these materials is an intellectual property of the mentioned company. Therefore, no information about them can be provided. Fixed volumes of each material were considered to evaluate each injection–extruding system and a combination of such materials. In this case, the combined operation was considered to construct simple forms of combined materials.

The two aspects of the project converge in the development of complex-structured foods, that is, foods in which the existence of two or more heterogeneous arranged materials is desirable. During this period, we have demonstrated the technical feasibility of using the printers and inks we have developed to manufacture complex-structured foods. These facts have been confirmed following the sequence of design steps considered in this research study, especially by combining the technical elements described above, ranging from the mechanical sections to the advanced automation system that coordinates both robotic devices.

## 3. Results

**Figure 5:**
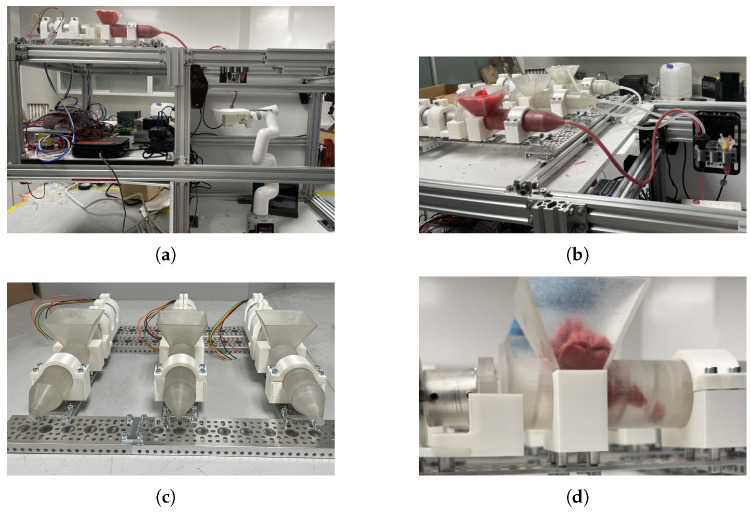
Composition of photos that detail the mechanical sections of the built food printer. (**a**) Implementation of the coordinated robotic configuration based on Cartesian–manipulator devices. (**b**) Injector configuration connected to the Cartesian system and extruder. (**c**) Detailed view of food ink container configuration. (**d**) Detailed view of the food ink screw and container.

The photographs shown in Figure 5 show the developed structure of our suggested food printer. The composition appearing in the mentioned figure demonstrates all significant sections in the food printer. Figure 5a shows a general photo of the food printer with all the technical components described in the methodological section. Figure 5b depicts the robotic system’s Cartesian section, showing the extruders’ relative position to observe the printing configuration at the end of the injector–extruder configuration. Figure 5c shows the robotic manipulator placed in the printing chamber. Finally, Figure 5d shows a general view of the printing system’s electronic section, including the proposed printer’s injector stage. All sections obtained correspond to the proposed configurations considered in the design stage.

Mechanical supporting sections were made of aluminum with profiles of 40 × 20 mm. Nema-34 stepper motors produced the motion of the Cartesian device that mobilized the gantry section that carried the extruder. In addition, the manipulator was instrumented with its own electronic interface. Both devices were connected as planned to the mini computer.

High pseudo-plasticity is expected for the selected food inks to extrude smoothly from the nozzle during the extrusion step. The experiments performed illustrate how apparent viscosity drastically decreased as the shearing rate increased, proving that all food inks were non-Newtonian fluids with high pseudo-plasticity. The same condition is observed with the pea protein ink, which showed a higher initial viscosity value with a lower slope when shear stress increases (above 1000 Pa s). The values of the power law are $K_{f}=42.11\pm 1.24\;Pa\cdot s$, n=0.4±0.06 with a yield stress of 0.34±0.04. A brief flow velocity analysis was performed through image processing using ImageJ software V1.0 to obtain the velocity at which the different food inks flow through the nozzle. The flow velocities are reported in Table 2.

**Figure 6:**
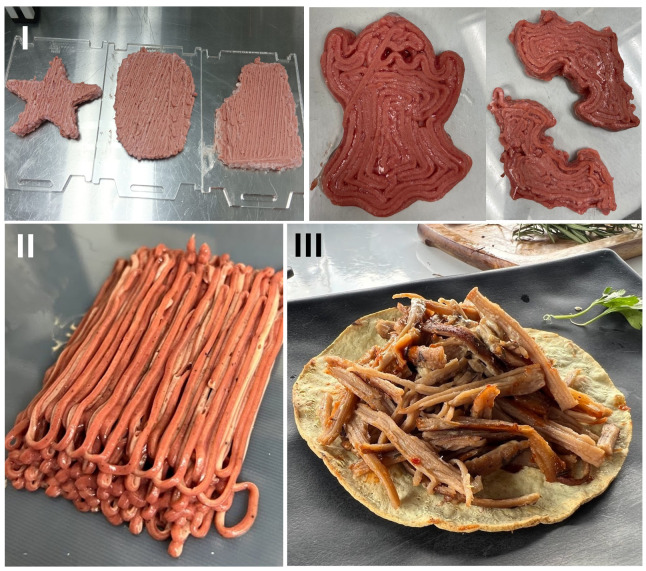
Examples of printed structures with multiple inks, each one placed in an individual channel extrusion system. (**I**) Planar-shaped forms with complex exterior edges. (**II**) Multilayer structures. (**III**) Cooked meat produced with the printer.

The selected ink seems to show a non-Newtonian (shear thinning) behavior. In this case, as analyzed in different studies, moving the food ink faster does make dragging the material more difficult, but not proportionately. Increasing the shear rate in several viscous liquids that undergo shear thinning makes things comparatively more straightforward because the viscosity decreases. This fact was confirmed by evaluating the food printer proposed in this study. Figure 6 shows different examples of printed structures based on vegetable-based meat. These shapes take advantage of all the rheological properties, possibly creating high aspect ratios such as curved surfaces that form concave sections, multilayered structures, and straight diagonalized planes.

The application of the food printer with a single but previously mixed multi-material showed the possibility of performing traditional planner-shaped structures with a small thickness. Several different shapes are depicted, showing the printing with a single layer of synthetic meat and several layers of the same material. These results confirm the possibility of creating structures with a single material, which shows the applicability of the printer to developing structures with complex shapes.

**Figure 7:**
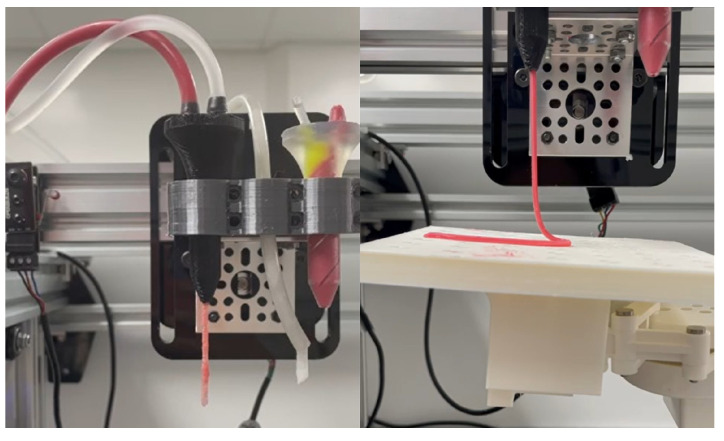
Composition of the multi-ink extrusion system using the synthetic meat and the proposed connective emulated tissue.

Figure 7 shows the configuration of the extruder section in the printer carrying different materials. This printing configuration demonstrates the deposition of mixed material in the final nozzle and the individual material that emerges with controlled flow. In addition, the same figure demonstrates the extrusion and deposition process of the mixed material over a planar surface, holding it at the end effector of the robotic manipulator. This characteristic is one of the contributions of the proposed robotic configuration, which can mobilize both sections in a complementary manner, as shown in the figure mentioned above.

**Figure 8:**
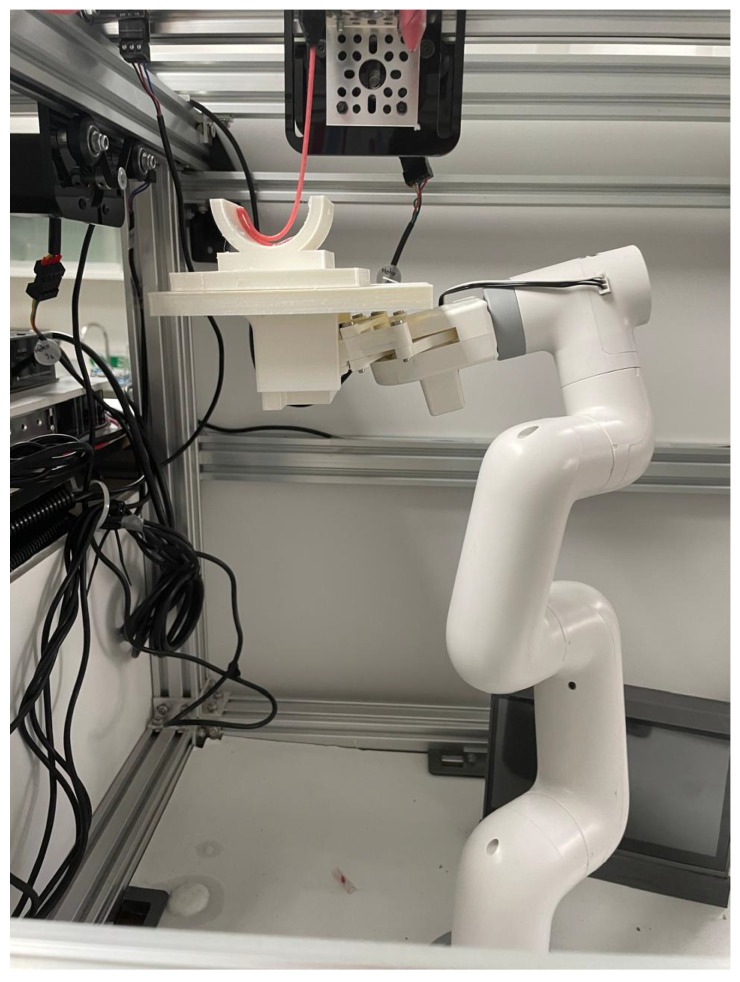
Example of curved surfaces in a 3D shape based on synthetic meat as extruded material.

Figure 8 demonstrates the application of the printer section that deposits mixed material on a curved surface, which the robotic manipulator mobilized. This printing strategy allows the creation of complex shapes using different food inks simultaneously and directly combined in the nozzle section of the printing system.

All of the previous images show that the use of the printing strategy with static mixing elements in the extruder section produces partial mixing of two edible viscous inks. The microarchitecture of goods such as beef, where muscle and fat tissue coexist but are not uniformly mixed but are still somewhat separated at the micro scale, is simulated by this partial mixing. Figure 8 displays the results of the multi-material 3D printing of a miniature curved-shaped form, where a fatty component and a protein component coexist steadily.

**Figure 9:**
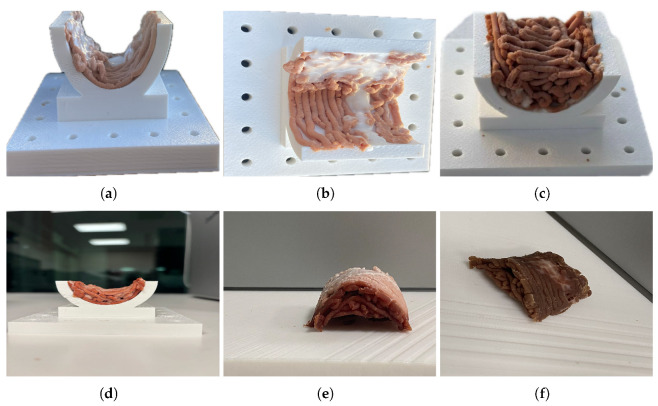
Composition of photos that detail the steps to print curved food. It can be observed that the curvature is preserved after demolding and that the multi-material deposition allows the mixing of food inks with different rheological properties, emulating the real mechanical and texture properties of meat. (**a**) Lateral view of the first layer’s multi-material deposition on the curved template. (**b**) Top view of the first layer’s multi-material deposition on the curved template. (**c**) Stacked curved printed layers. (**d**) Lateral view of the final curved printed food. (**e**) Final curved printed food removed from the template. (**f**) Final product, cooked curved printed food.

Figure 9 shows the sequence of stages needed to create a multi-material piece of synthetic food. The sequence demonstrates different stages in creating the synthetic three-dimensional structure that adjusts to the curved surface corresponding to the plastic supporting structure. The figure also exemplifies the possible geometries to be printed, elevating the complexity and versatility of current 3D food printers. It is demonstrated that curvature is retained and that the proposed method for mixing food inks widens the options for producing a variety of foods, controlling aspects such as texture, consistency, and nutritional value.

## 4. Discussion

The results show that the prepared food inks are within the viscosity range to be extruded according to [15], and can remain stable after printing. The injection system shows the advantage of easy material refill, and the screw conveyor system allows the extrusion of materials with different rheological properties without mechanical modifications. Moreover, integration with the Kenics Static mixer allows for the simultaneous mixing and depositing of multiple materials, which modifies the rheological properties of the food inks, as observed in the change of mass flow velocity. Moreover, regulating the relative extrusion velocity for each material and mixing at the nozzle level allows the material characteristics to be controlled according to the food design requirements. The proposed design of the multi-extruding system can be adapted to different materials, ranging from classical hydrogels to clay-based materials. This flexibility is an additional contribution that is considered crucial in the proposed printing system.

The aggregation of the robotic arm allows the construction of curved shapes, which is not a common characteristic in 3D printers, even for materials other than those used in food production. In particular, the developed printer system provides a strategy to regulate the motion of both the extruder and the deposition surface. This approach improves the possibilities for extruding printer devices that can be used for all possible materials that can be developed using additive manufacturing strategies [4]. Moreover, the proposed device configures a hyper-redundant robotic configuration that provides sufficient degrees of freedom to create complicated shapes for food inks. In addition, the level of automation introduced in this paper can be extended to modify the characteristics of the final additive-manufactured object, an additional benefit of the design strategy considered here.

## 5. Conclusions

The study demonstrates significant progress in the development of realistic artificial foods by integrating multiple biomaterials in a controlled and ordered manner using advanced additive manufacturing technologies. The fully automatized food printer uses a unique combination of Cartesian and multi-ink robotic systems to handle various food inks with varying rheological properties, expanding the versatility and precision of food printing. The development of the printer follows a structured mechatronic design strategy, which ensures its reliability and functionality through robust instrumentation and automation systems. Implementing an adaptive controller and coordinated motion based on G-code improves motion precision, enabling the creation of complex and intricate food structures. Testing with commercial food inks confirms the printer’s capability to produce complex three-dimensional shapes and multi-material structures, showcasing its potential for synthetic tissue creation and diverse applications in food manufacturing. The study highlights the potential for this technology to modify synthetic food production, offering new possibilities for creating customized, nutritious, and aesthetically appealing artificial foods.

Current limitations of the proposed multi-material 3D food printer include the need to characterize each material used, in the sense that the flow velocity and overall rheology characteristics of the ink need to be known since they are necessary to set the appropriate RPM of the screw conveyor system. In addition, depending on the viscosity of the ink and the size of the piece to be printed, the printing time can be slow due to the time needed to travel across the plastic hose. Additionally, the printing of curved pieces is currently limited to pre-established curved shapes since 3D PLA-printed templates are needed as printer support. Therefore, specific templates should be prepared according to the desired shape.

Future work arising from the results of this study includes the option of developing more complex multi-material structures with controlled positions and orientations. In the same way, based on the technological advances presented in this study, it is reasonable to produce curve-shaped artificial foods with improved nutritional characteristics.

## Figures and Tables

**Table 1:** Mechanical components.

System	Injectors	Cartesian	Manipulator
	3 extruders	X-Y robots	6 revolute joints
Components	Ink container	NEMA 34 stepper motor	Stepper motor
	food ink screw		
	12 DC Motor		
End effector	Hose as conduit medium	Kenix static mixer (extruder)	Mobile food printer’s base

**Table 2:** Food ink volumetric flow through the nozzle.

	Pea Protein Ink	Pea Protein InkMixed with Vegetable Fat	Pea Protein Ink with Synthetic Connective Tissue
mL/s (mean)	4.62 × $10^{-4}$	6.78 × $10^{-4}$	2.55 × $10^{-3}$
S.D.	0.0009	0.0013	0.0024

## Data Availability

All data including simulation files will available upon appropriate request to the corresponding author.

## Abbreviations

The following abbreviations are used in this manuscript:

3D	Three-dimensional
DC	Direct current
PMAC	Programmable multi-axis controller
PWM	Pulse width modulation
TE	Tracking error
RPM	Revolutions per minute
PMAC	Multi-axis controller
FDM	Fused deposition modeling
